# Maintenance of core temperature in SCUBA divers in cold water: contributions of anthropometrics, suit type, and sex

**DOI:** 10.1007/s00421-025-05961-5

**Published:** 2025-09-04

**Authors:** Tucker Orman, Karleigh E. Bradbury, Tim Grosshennig, Makayla Perez, Fabian N. Möller, Željko Dujić, Andrew T. Lovering

**Affiliations:** 1 Department of Human Physiology, University of Oregon, 1025 University St., 218 Pacific Hall, Eugene, OR 97405 USA; 2 United States Army Research Institute of Environmental Medicine, Natick, MA 01760 USA; 3 Department of Psychology and Sports Science, University of Innsbruck, Innsbruck, Austria; 4 Aerospace Physiology Laboratory, Massachusetts Institute of Technology, Cambridge, MA 02139 USA; 5 Department of Integrative Physiology, University of Split School of Medicine, Split, Croatia

**Keywords:** Thermoregulation, Wetsuit, Cold water immersion, Body temperature

## Abstract

Maintenance of core temperature (Tc) is vital for health and physiological function while SCUBA diving in cold water, but there is little research investigating the influence of anthropometrics, suit type, and sex on the rate of change in Tc during real-world diving conditions. We measured the rate of change in Tc (telemetric pill) and thermal sensation (Ts; Young questionnaire) in 61 participants (31 female) before and after non-decompression SCUBA dives using open circuit apparatus breathing air at varied depths and durations in cold water (~ 10 °C). Twenty-two participants wore drysuits (10F), and 39 participants wore wetsuits (21F). There was a significant effect of suit type on the rate of change in Tc, with those in wetsuits having a greater decrease in Tc than those in drysuits. However, there was no effect of suit type on the rate of change in Ts. In wetsuit and drysuit groups, there were significant associations between Tc/min and BSA/BM, BMI, and BM. Estimated body fat % (BF%) was significantly associated with the rate of change in Tc in the wetsuit group only. When separated by sex, there were significant associations with all the anthropometric variables and the rate of change in Tc in the female participants, apart from BMI, but only with BM in the wetsuit males. These results suggest that drysuits offer greater thermal protection compared to wetsuits in 10 °C water, and anthropometrics should be considered when selecting the degree of thermal protection, especially for female divers.

**Graphical abstract:**

**Figure:**
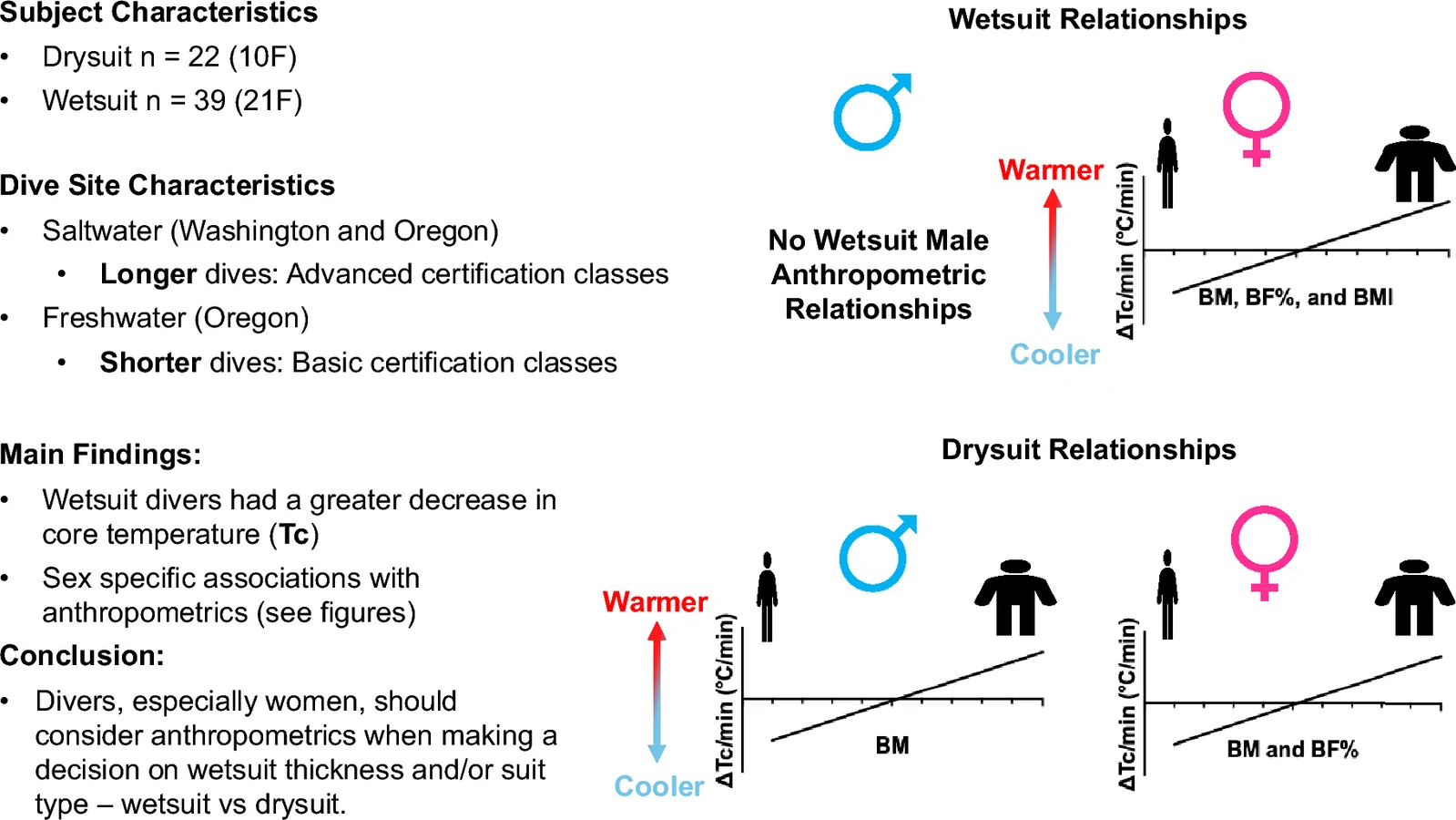

## Introduction

There are approximately 6 million active SCUBA divers worldwide, and 40% of them are women (*2018 Consumer Household Analysis: Open Water Divers*, 2018). SCUBA divers are often exposed to water temperatures below the thermoneutral range for humans of 34–35 °C (Castellani and Young 2016; Pendergast and Lundgren 2009). However, maintaining core temperature in that thermoneutral temperature is more difficult in water immersion relative to air (Pallubinsky et al. 2019) due to the 25-fold greater conductivity of water compared to air (Cappaert et al. 2008). This higher conductivity has important implications for thermoregulation and the maintenance of core temperature (Tc) in SCUBA divers. In most healthy humans, Tc is regulated at ~ 37 ± 0.5 °C (Morrison and Nakamura 2019), even in cold environments, due to heat production and conservation mechanisms. However, if these physiological mechanisms are not effective at maintaining Tc, decreases in Tc can have numerous negative consequences for divers, especially in cold water (Castellani and Young 2016; Kelly et al. 2022; Muza et al. 1988; Yurkevicius et al. 2022). Consequences include, but are not limited to, decreases in cerebral blood flow (Gibbons et al. 2020) and neurological excitability (Hurrie et al. 2022; Talebian Nia et al. 2023). These changes can affect executive function and higher order processes like decision-making and problem-solving (Sudo et al. 2022; Tempest et al. 2017), which poses a significant risk to those who SCUBA dive, as clear thinking and unimpaired physiological function are critical when diving. Thus, the U.S. Navy has defined exposure limits as a decrease in Tc of 1 °C (Beatty and Berghage 1972; Doubt 1996).

Other factors that contribute to the maintenance of Tc during cold-water immersion (CWI) or SCUBA diving include behavioral thermoregulation, defined as increased physical activity and temperature-adapted suit selection (Doubt 1996; Lafère et al. 2021), as well as anthropometric characteristics. Total body mass (BM), body surface area (BSA), body surface area to mass ratio (BSA/BM), body mass index (BMI), and body fat % (BF%) can influence Tc perturbation experienced during CWI. Individuals with higher BMs, higher BMIs, and lower BSAs have all been shown to have smaller Tc decreases following CWI without the protection of wetsuits or drysuits (Castellani and Young 2016; Gagge and Gonzalez 2011; Toner and McArdle 2011; Zhang et al. 2023). The previous CWI literature has addressed primarily males and females without thermal protection (i.e., wetsuits or drysuits). For example, in the work by McArdle and colleagues, participants wore nylon swimsuits only (McArdle et al. 1984a, b). Similarly, in those few studies where subjects have work thermal protection to CWI, the participants have been mostly/only male subjects wearing wetsuits or drysuits in water conditions from 0 to 5 °C, e.g., Lafère et al. (2021) and Lundell et al. (2019). Suit selection is also an important factor to consider in the setting of cold-water diving. Drysuits effectively keep cold water from directly contacting the skin outside of the obligatory head and neck regions but rely on the material of the drysuit (i.e., either trilaminate fabric, compressed/crushed neoprene, or a combination) and thermal undergarments to keep divers warm. Conversely, neoprene wetsuits allow water to contact the skin, and the thickness of the wetsuit is adjusted depending on the water temperature to provide adequate thermal protection. While these decisions on suit type, thickness, and undergarment selection are typically made by individual divers and diving outfitters, they are often done so without the additional consideration of the anthropometric characteristics of each individual diver.

Work from our lab has recently reported that anthropometric factors and wetsuit thickness are important for maintaining Tc during SCUBA diving in those wearing wetsuits (Bradbury et al. 2024). However, there are a few limitations and knowledge gaps in that study that we wish to build upon in the current work. First, that study included very few females. Therefore, a current knowledge gap is whether females present different rates of change in Tc or Ts after SCUBA diving. Second, the study was done in water temperatures ranging between 17 and 26 °C (bottom and surface temperatures, respectively). Whether or not these relationships between anthropometric characteristics and Tc are still present in colder water temperatures is unknown. In addition, while it is commonly accepted within the SCUBA diving community that drysuits provide better protection from cold-water exposure than wetsuits, only indirect measures of Tc via high-resolution thermal infrared imaging of cervical-supraclavicular skin have been reported in two different groups of men wearing either a wetsuit or drysuit. In that study, those wearing wetsuits had a lower Tc than those wearing drysuits after a ~ 35–40 min dive in 5 °C water (Lafère et al. 2021). However, whether those who choose to wear a drysuit vs. a wetsuit are better able to maintain Tc requires confirmation through direct (internal) core temperature measurements.

Therefore, the purpose of this study was to extend our previous research and determine the influence of the following factors on Tc and thermal sensation (Ts) during SCUBA diving in colder water dives (10 °C): (1) suit type (wetsuit vs. drysuit), (2) sex assigned at birth, and (3) anthropometric characteristics. We hypothesized that: (1) those wearing wetsuits would have a greater decrease in Tc and Ts than those wearing drysuits; (2) anthropometrics within each sex would primarily drive rate of change in Tc and Ts, independent of suit type; and, (3) those with high BMIs, BMs, and calculated BF% would have the smallest rate of change in Tc, while those with the highest BSA/BM ratio would have the greatest rate of change in Tc, independent of suit type.

## Methods

### Ethical approval

This study received approval from the University of Oregon’s Office for the Protection of Human Subjects (STUDY00000951). Each participant was given documents outlining the study and provided written approval prior to participating in the study. All experimental procedures were conducted in accordance with the *Declaration of Helsinki 2013*.

### Participants

Sixty-one divers participated in cold water dives (10.6 ± 2.0 °C) between October 2023 and February 2024. Participants were recruited from the University of Oregon’s courses for basic and advanced SCUBA certifications, adhering to the Professional Association of Diving Instructors (PADI) guidelines. The Advanced Certification divers and dive instructors (*n* = 46 divers; 23 M, 23 F) dove in saltwater in Hood Canal off the coast of Lilliwaup, WA, USA, and off the coast of Florence, OR, USA. The Basic Certification divers (*n* = 15 divers; 7 M, 8 F) dove at Woahink Lake in Florence, OR, USA. Thirty-nine divers wore wetsuits (18 M, 21 F), and twenty-three wore drysuits (12 M, 10 F). All wetsuit divers had the same core and peripheral wetsuit thickness, comprising 7 mm farmer johns, 7 mm shorty long sleeves, and wore 5 mm gloves, 5 mm hoods, and 6 mm booties. The decision to use these thicknesses of suits and peripherals is the standard rental equipment used in the Pacific Northwest for dives in the 10–12 °C waters. Before their dives, participants signed an informed consent form confirming they were in healthy condition, free of major gastrointestinal issues, and not currently undergoing hormone replacement therapy. Anthropometric information (height and weight), sex assigned at birth, and age were collected on all participants (Table 1).

**Table 1:**
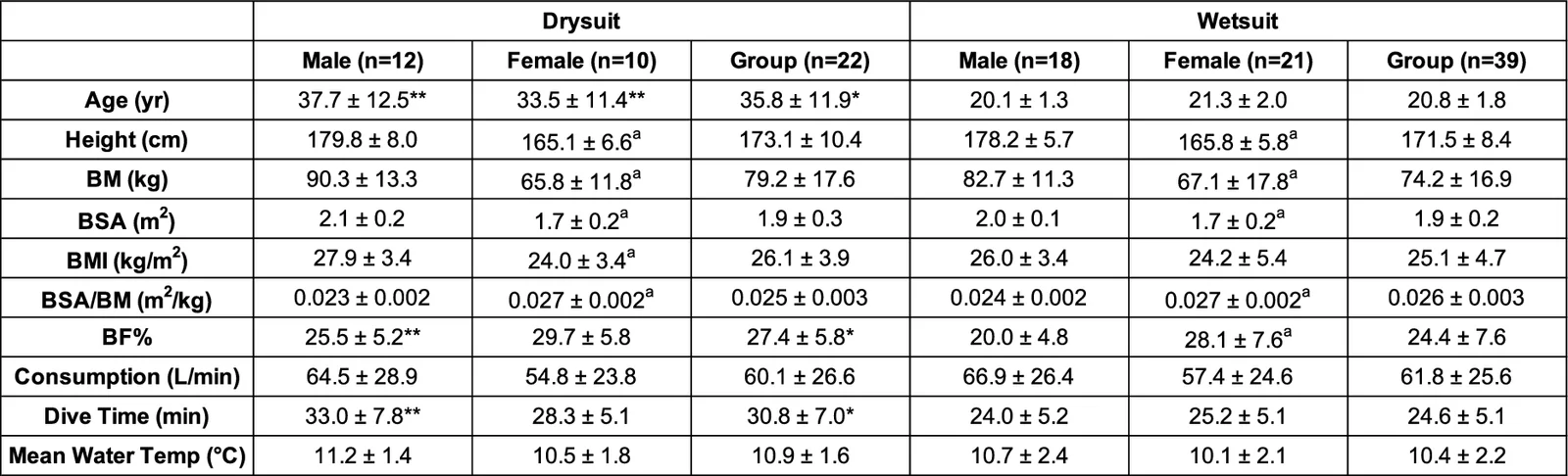
Anthropometric, air consumption, dive time, and average water temperature information on drysuit and wetsuit divers, separated for sex

*Indicates suit type difference for each group, **indicates sex by suit type difference

^a^Indicates sex difference within a group

### Data collection

Each participant ingested a telemetric core temperature pill (Jonah core body temperature capsule; Mini Mitter, Bend, OR, USA) on the night before their dive (~ 10 h prior to the dive). Divers would be expected to have some increase in Tc just before entering the water because they must don their suits before putting on their weight belts and subsequently their diving gear, which is all connected to their buoyancy compensation device (BCD), which contains: additional weight in the ditchable pockets, regulators, pressure gauge, and tank. For this reason, we took their Tc after donning all their gear but immediately before entering the water to control for activity and take into consideration their actual Tc immediately before diving as opposed to their resting Tc before donning their gear. Immediately before entering and after exiting the water, each participant’s tank pressure, Tc, and Ts were collected with a single measurement at each time point. Participants were asked to report their Ts using a visual scale from 0 (unbearably cold) to 8 (unbearably hot) (Young et al. 1987). Tank pressure was assessed with individual mechanical pressure gauges. Total dive time, mean water temperature, and mean depth were assessed via the group leader’s dive computer. Minimal variability in water temperature suggested little to no thermocline in the dive locations, which is expected in the areas where dives took place (Table 2).

**Table 2:** Average water temperature and depth for saltwater (Hood Canal, Washington and Florence, Oregon), freshwater (Woahink Lake, Florence, Oregon), and combined dive sites

	Saltwater	Freshwater	Overall
Mean water temp (°C)	10.2 ± 1.6	11.7 ± 2.7^a^	10.6 ± 2.0
Mean depth (m)	10.9 ± 3.9	5.6 ± 1.1^a^	9.6 ± 4.1
Dive time (min)	28.0 ± 6.8	23.4 ± 3.8^a^	26.9 ± 6.5

^a^Indicates difference between sites

### Calculations

To account for variation in metabolic rate and its influence on Tc maintenance, total air consumption (L) and minute ventilation (VE; L/min) were calculated as before (Bradbury et al. 2024; Möller et al. 2023)

$${\text{Total air consumption}}\left( {\mathrm{L}} \right) = \left( {\Delta {\mathrm{Ptank}}*{\mathrm{Vtank}}} \right)/{\mathrm{Pdepth}}$$

$${\mathrm{V'E}}\left( {{\mathrm{L}}/{\mathrm{min}}} \right) = {\text{Total air consumption}}/{\text{Dive time}}$$

V'E was calculated using pre–post-dive tank pressure and mean depth recordings from dive computers. ∆Ptank (kPa) is defined as the difference in tank pressure pre- vs. post-dive. V tank (L) is the volume of the diver’s tank. Pdepth (kPa) is the pressure at the mean depth during the dive.

The rate of change in Tc per minute (∆Tc/min) was calculated using pre–post-telemetric pill measurements.

$${\text{Rate of Tc change }}\left( {^\circ {\mathrm{C}}/{\mathrm{min}}} \right){\text{ was calculated as }}\left( {{\text{Post Tc}} - {\text{Pre Tc}}} \right)/{\text{Dive time}}$$

Each diver’s BF% was estimated using the equation below (Jackson et al. 2002).

$${\text{Estimated BF}}\% = \left[ {\left( {{1}.{39}*{\mathrm{BMI}}} \right) + \left( {0.{16}*{\mathrm{Age}}} \right) - \left( {{1}0.{34}*{\mathrm{Sex}}} \right) - {9}} \right];{\text{with male}} = {\text{1 and female}} = 0.$$

### Statistical analysis

GraphPad PRISM Version 10.2.3 was used for all graph-making and statistical analysis. Unpaired two-tailed *t* tests assuming equal variance were run to compare depth and temperature differences between dive sites (freshwater vs. saltwater) and both the rate of change in Tc/min and the rate of change in Ts/min between sexes and suit types. A two-way ANOVA with multiple comparisons using the uncorrected Fischer’s LSD was used to analyze the effects of sex and suit type on age, body mass, height, BSA, BSA/BM, BMI, air consumption, dive time, and average water temperature. Linear regressions compared the rate of change in Tc/min to BSA/BM, BM, and BMI in wetsuit and drysuit divers. Statistical significance was set at *p* < 0.05. Using G*Power (v3.1.9.6), we performed a post hoc power analysis with our rate of change in Tc/min data between wetsuit and drysuit wearers and determined that we had a power of 1.0.

## Results

### Participants

Participants’ anthropometric information, air consumption, dive time, and average water temperature were all separated by suit type and further between sexes, presented in Table 1. Males and females wearing drysuits were significantly older than those wearing wetsuits (within the same sex; *p* < 0.05). Within both the wetsuit and drysuit groups, the males were taller and had higher BM and BSA, but lower BSA/BM than the females (*p* < 0.05). Males wearing drysuits had a significantly higher BMI than females wearing drysuits (*p* = 0.0235). However, there was no difference in BMI between males and females wearing wetsuits (*p* = 0.2309, Table 1). Calculated BF% was greater in drysuit divers compared to wetsuit divers (p = 0.0356, Table 1). Calculated BF% was greater in males wearing drysuits compared to males wearing wetsuits, and females wearing wetsuits had a greater BF% than males wearing wetsuits. There were no differences in calculated air consumption between suit type or sex (*p* > 0.05, Table 1). Similarly, there were no differences in water temperature between suit type nor sex (*p* > 0.05, Table 1). However, male drysuit participants dove for longer durations than male wetsuit divers (*p* < 0.0001, Table 1). Given this variation in dive duration, we present both the rate of change in Tc and Ts as a function of time to make these comparable. There were no sex-related differences in dive time for wetsuit subjects (*p* > 0.05) or between suit types for female divers (*p* > 0.05, Table 1).

### Dive sites

The water temperatures and depths for the two dive sites are presented in Table 2. Dives in saltwater were, on average, colder (*p* = 0.0158), deeper (*p* < 0.0001), and longer (*p* = 0.0159) than those in freshwater. However, we saw no differences in the rate of change in Tc/min nor the rate of change in Ts/min between wetsuit divers that dove in saltwater compared to freshwater (*p* > 0.05, data not shown). All drysuit divers dove in saltwater.

### Influence of suit type on uncorrected thermoregulatory variables

Pre-dive Tc was greater in females wearing a drysuit, but there were no differences in post-dive Tc before correcting for dive time (Table 3). Tc uncorrected for dive time was only significantly decreased in females wearing wetsuits (Table 3). Ts significantly decreased in all divers, with the greatest decrease in females wearing wetsuits (Table 3).

**Table 3:** Absolute values for pre-dive and post-dive Tc and Ts in drysuit and wetsuit divers, separated for sex

	Drysuit (*n* = 22)				Wetsuit (*n* = 39)			
	Male (*n* = 12)		Female (*n* = 10)		Male (*n* = 10)		Female (*n* = 21)	
	Pre-dive	Post-dive	Pre-dive	Post-dive	Pre-dive	Post-dive	Pre-dive	Post-dive
Tc (°C)	37.6 ± 0.3	37.7 ± 0.4	38.0 ± 0.3^a﻿^	37.9 ± 0.3	37.5 ± 0.2	37.2 ± 0.4	37.8 ± 0.3	37.2 ± 0.6^b^
Ts (a.u)	5.0 ± 1	3.3 ± 1^b^	4.9 ± 0.9	3.1 ± 1.4^b^	5.0 ± 0.7	3.5 ± 1.2^c^	4.4 ± 1.0	2.4 ± 1.2^b^

Note that post-dive Ts and Tc do not control for variations in dive times; see figures for values corrected for dive time

^a^Indicates sex difference

^b^Indicates dive time difference (pre- vs. post-dive)

^c^Indicates sex and dive time difference

### Time-corrected thermoregulatory variables

**Fig. 1:**
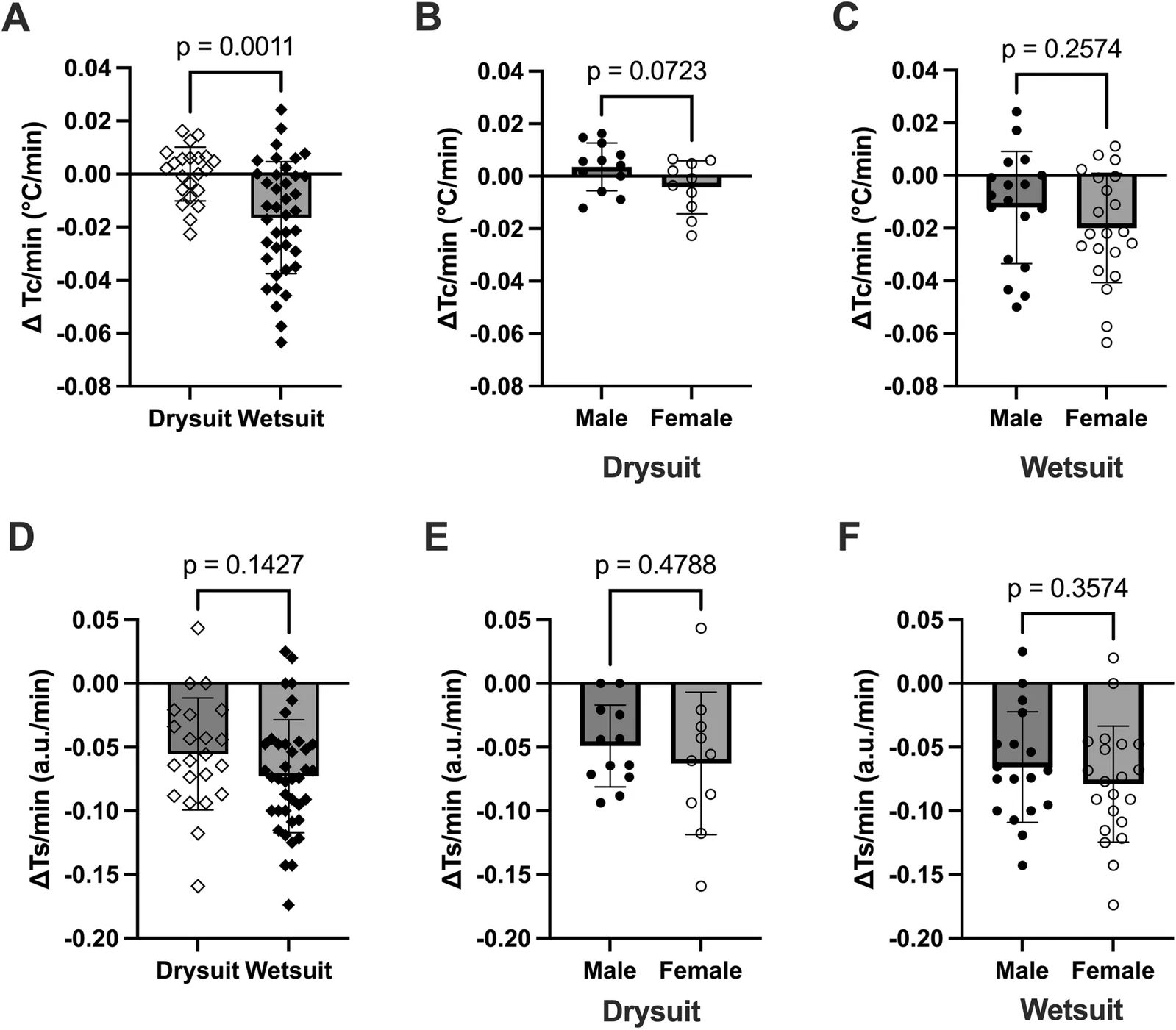
Rate of change in core temperature (∆Tc/min) (**A**) and thermal sensation (∆Ts/min) (**D**) comparing wetsuit and drysuit divers. Drysuit sex-specific rate of change in core temperature (∆Tc/min) (**B**) and thermal sensation (∆Ts/min) (**E**). Wetsuit sex-specific rate of change in core temperature (∆Tc/min) (**C**) and thermal sensation (∆Ts/min) (**F**). Black diamonds denote wetsuit participants and white diamonds denote drysuit participants (**A** and **D**). Black circles denote male participants, and white circles denote female participants (**B**, **C**, **E**, and **F**)

Again, given that the dives had varied durations, we analyzed the Tc and Ts data as changes in Tc and Ts over dive time. There was an effect of suit type on the rate of change in Tc/min (*p* = 0.0011), with those in wetsuits having a greater rate of change in Tc/min (− 0.02 ± 0.02 °C/min) than those in drysuits (0.00 ± 0.01 °C/min) (Fig. 1A). There were no differences in the rate of change in Tc/min between sexes, regardless of suit type (*p* > 0.05, Fig. 1B and 1C).

Further, there was no effect of suit type with respect to the rate of change in Ts/min (*p* > 0.05, Fig. 1D). Similar to the rate of change in Tc/min, there were no differences in the rate of change in Ts/min between sexes, whether the divers wore wetsuits or drysuits (*p* > 0.05, Fig. 1E and F).

### Relationships between anthropometrics and rate of change in core temperature

**Fig. 2:**
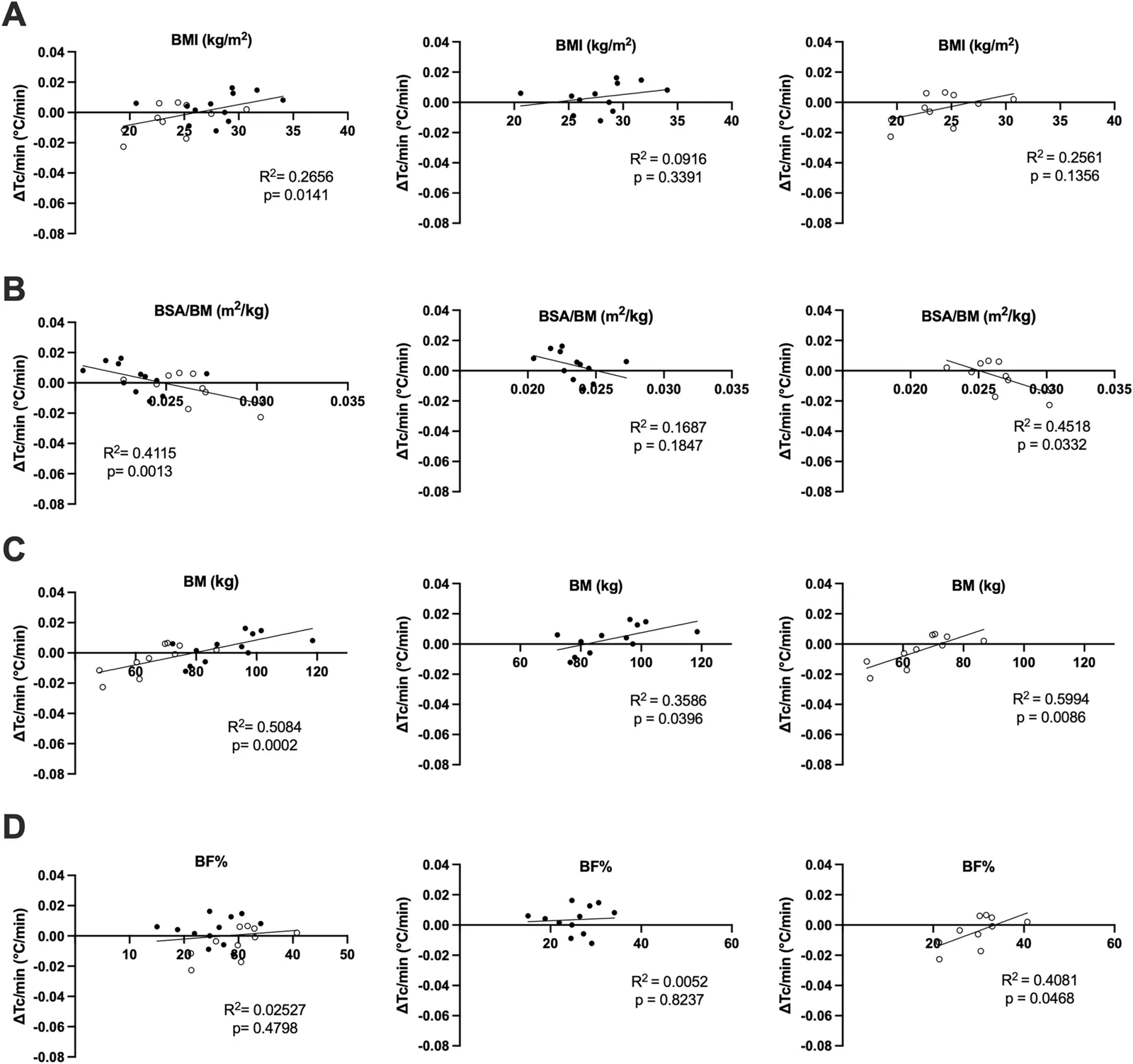
Correlations between anthropometric variables (**A** BMI, **B** BSA/BM, **C** BM, **D** BF%) and rate of change in core temperature (∆Tc/min) in drysuit divers as a group and separated for sex. Black circles denote male participants, and white circles denote female participants

In the group of drysuit divers, the BSA/BM was negatively correlated with the rate of change in Tc/min (*p* = 0.0013, *R*^2^ = 0.4115), whereas both BMI (*p* = 0.0141, R^2^ = 0.2656) and BM (*p* = 0.0002, *R*^2^ = 0.5084), were positively correlated with the rate of change in Tc/min. (Fig. 2). In the group combined, BF% was not significantly correlated with the rate of change in Tc/min (Fig. 2). Within each sex, only BM was significantly associated with the rate of change in Tc/min (*p* = 0.0396, *R*^2^ = 0.3586) in males. In contrast, BSA/BM, BM, and BF% were all significantly associated with the rate of change in Tc/min, with the highest R^2^ value observed between BM and the rate of change in Tc/min (*p* = 0.0032, *R*^2^ = 0.6371) in females wearing drysuits (Fig. 2).

**Fig. 3:**
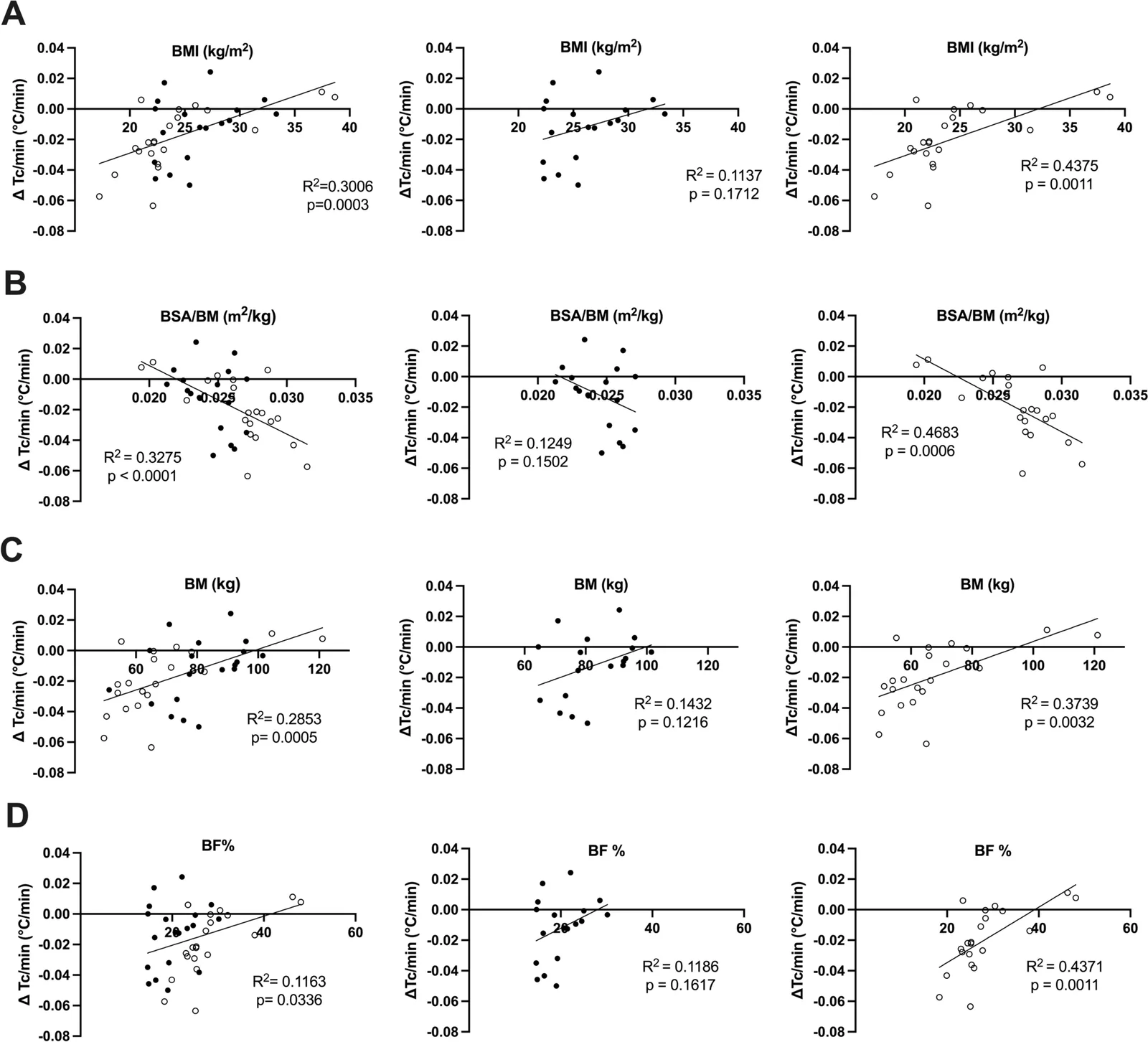
Correlations between anthropometric variables (**A** BMI, **B** BSA/BM, **C** BM, **D** BF%) and rate of change in core temperature (∆Tc/min) in wetsuit divers as a group and separated for sex. Black circles denote male participants, and white circles denote female participants

Wetsuit divers presented similarly to the drysuits, with BSA/BM negatively correlated with the rate of change in Tc/min (*p* < 0.0001, *R*^2^ = 0.3275) and BMI (*p* = 0.0003, *R*^2^ = 0.3006). BM (*p* = 0.0005, *R*^2^ = 0.2853) and BF% (*p* = 0.0336, *R*^2^ = 0.1163) were positively correlated with the rate of change in Tc/min (Fig. 3). Within each sex, no anthropometric variables were significantly associated with the rate of change in Tc/min in males. However, BMI, BSA/BM, BM, and BF% were all significantly associated with the rate of change in Tc/min, with the highest R^2^ value observed between BSA/BM and the rate of change in Tc/min (*p* = 0.0006, *R*^2^ = 0.4683) in females wearing wetsuits (Fig. 3).

### Other

We found no significant associations with age and the rate of change in core temperature within either suit type (*p* = 0.9753 for wetsuits and *p* = 0.8321 for dry suits), data not shown.

## Discussion

The current study directly measured Tc and Ts in 22 drysuit and 39 wetsuit divers before and after SCUBA diving in ~ 10 °C water, making this the largest study to date with these measures that included females. We found that: (1) compared to drysuit divers, wetsuit divers had a greater rate of change in Tc/min, but both groups had a similar rate of change in Ts/min; (2) there were no sex-based differences in the rate of change in Tc/min or the rate of change in Ts/min within each suit type; (3) there were significant associations between the rate of change in Tc/min and BSA/BM, BMI, and BM within both suit types as a group; however, when examining sex-specific anthropometric associations within a suit type, the associations remained in females, except for BMI, and were all absent in the males with the exception of BM in males wearing drysuits. Sex-specific associations revealed that BF% was significantly associated with the rate of change in Tc in females in both suit types, while associations for the whole group were only found in wetsuit divers. There were no sex-specific relationships between BF% and the rate of Tc change in male divers. We discuss the implications of these findings in detail below.

While both wetsuits and drysuits help to keep the diver warm during dives, the mechanisms and extent to which they do so differ. Wetsuits have variable thicknesses for thermal protection but also expose the diver to a layer of water surrounding the diver that must be heated by the body. Depending on the temperature of the water, this results in a loss (most common) or gain of body heat to the water in the wetsuit due to the thermal gradient (Gu et al. 2023). Depending on water temperature, physical activity, and anthropometric characteristics, the loss of body heat to the surrounding water in the wetsuit will lead to decreases in Tc, as previously reported (Bradbury et al. 2024; Lafère et al. 2021). Conversely, drysuits, by design, eliminate the entrance of the cold water into the suit and provide a physical barrier between the diver and the external environment, but require the diver to select the appropriate thermal undergarments for warmth. Except for the head and neck region, the majority of a drysuit diver’s skin is not exposed to the cold water during dives. Therefore, it can be expected that drysuit divers would lose less heat and, therefore, experience smaller changes in Tc during the dives. Due to these differing protective properties of each suit type, the greater rate of change in Tc/min seen in those wearing wetsuits vs. drysuits was expected, as previously reported using indirect measures in males only (Lafère et al. 2021). However, until this study, differences in the rate of change in Tc/min using direct measures between males and females wearing either drysuits or wetsuits have not been previously reported.

Interestingly, in our study, we found that there were no differences in the rate of change in Ts/min between the two suit types despite significant differences in Tc/min. When uncorrected for dive time, we found that male and female drysuit divers had similar pre- and post-dive Ts. Conversely, when uncorrected for dive time, despite male and female wetsuit divers having similar pre-dive Ts, the females had significantly lower Ts post-dive. It is established that skin temperature (Tsk) is related to Ts (Song et al. 2024), and the majority of the skin of the wetsuit divers is indirectly exposed to the cold water. Because of this, we hypothesized and expected that those wearing wetsuits would have a greater rate of change in Ts/min during the dive, given the greater thermal conductivity of water relative to air. While the wetsuit allows indirect water contact with the skin, the water is also warmed by body heat throughout the dive, unlike the water that surrounds those wearing drysuits, which remains cold. It has previously been reported that Tsk decreases in those wearing drysuits in arctic conditions (Lundell et al. 2019). Thus, it is possible that the drysuit divers felt as cold as the wetsuit divers despite differences in the rate of Tc change. Importantly, we did not make measures of Tsk in this study as Tsk cannot be measured at depth in water with existing Tsk technology. Future investigations should measure Tsk, Tc, and Ts during SCUBA diving to gain a greater understanding of the interaction between these variables.

It is well established that a component of thermoregulatory responses in the cold is behavioral thermoregulation, which includes increasing physical activity to increase metabolic heat production, thus aiding in the maintenance of Tc. Like our previous investigation in the field (Bradbury et al. 2024), we could not standardize physical activity during the dive, but we did so before the participants entered the water. Participants were asked to don their gear once at the dive site, which they did immediately prior to the pre-dive Tc measurement. Post-dive Tc was taken immediately after the participants exited the water, before participants removed their gear and equipment. To account for the lack of standardization of physical activity during the dive, we calculated the volume of air consumed (L) and minute ventilation (V'E) of all SCUBA divers. We found no difference in air consumed during the dive, which is an estimate of physical activity and/or metabolic rate, between those wearing wetsuits and drysuits, nor between sexes. Because those wearing wetsuits did not feel colder than those wearing drysuits, as indicated by no differences in Ts, it is possible that the wetsuit wearers did not feel the need to increase their physical activity (and therefore heat production) to stay within a tolerable comfort level during the dive. The lack of difference in inferred metabolic rate between the suit types also helps confirm that drysuits provide better thermal protection and less heat loss.

Our investigation was first to examine sex differences in Tc and Ts among individuals wearing wetsuits and drysuits during SCUBA dives. We found no differences between males and females in the rate of change in Tc/min or rate of change in Ts/min, regardless of suit type, which may be surprising given that we found significant differences in anthropometrics between males and females within each group and highlights the need to do studies in both men and women. The physiological response to, and changes in body temperature from, cold exposure are multifactorial. With cold exposure, peripheral and central thermoreceptors sense the reductions in temperature, and physiological responses such as vasoconstriction act to reduce heat loss, and shivering acts to increase thermogenesis. In the current study, our participants also wore thermal protection in the form of drysuits or wetsuits to minimize heat loss during the dive. In combination, differences in sensitivity of peripheral and central thermoreceptors, physiological responses, anthropometrics, and thermal protective outerwear may have all acted to offset each other, resulting in the lack of sex difference in the Tc change. We discuss some of these varying parameters below as they pertain to the observed sex differences in some cases and lack of sex differences in other cases.

In one study on cold-water immersion (in 20, 24, and 28 °C) where women were included, the participants did not wear thermal protection and had very low body fat percentages (McArdle et al. 1984a, b). In these studies, seven of the eight female subjects had between 15 and 25% body fat (Fig. 4 of that paper). From those studies in women with very low body fat, it was concluded that when controlling for body fat during a 1 h bout of arm-ergometer exercise in cold water in participants wearing nylon swimsuits only, there were no sex differences in the change in Tc (McArdle et al. 1984a, b). Nevertheless, these studies would suggest that when not wearing protective thermal clothing, the lack of a difference in Tc during exercise in cold water was due to the increased heat production, but also due to the increase in subcutaneous fat mass in the females, increasing insulation in the areas where heat production was the greatest. Similarly, a lower BF% and increased BSA/BM ratios lead to a greater decrease in Tc under CWI conditions (Castellani and Young 2016; Gagge and Gonzalez 2011; Toner and McArdle 2011). Increased BSA/BM ratios allow for enhanced conductive heat loss due to increased body surface area in contact with the colder environment, whereas decreased BSA/BM ratios and greater BMIs usually correlate with higher BF% and greater insulation relative to the surface area for heat dissipation.

Conversely, in the current study, most of the women were above 25% BF, and equally important, they were wearing thermal protection in the form of either wetsuits or drysuits. Thus, in women wearing thermal protection with greater BF% than has been investigated previously, we found sex differences in our associations with anthropometrics. In more recent studies in women with body fat percentages similar to those in the current study, selective skin cooling revealed that even when controlling for BF%, sex differences persisted, prompting the authors to speculate, as we have, that morphology and body composition are important factors with cold exposure (Dumont et al. 2022).

Why these anthropometric associations were not found in males in the current study is unknown. However, our males had a significantly greater muscle mass than the females, and muscle mass accounts for the majority of cold-induced thermogenesis (u Din et al. 2016). Thus, a greater muscle mass in our males, combined with the appropriate thermal protection, may have offset the effect of anthropometrics on maintaining core temperature during cold-water immersion in our study.

In the current study, our participants wore thermal protection in either the form of a wetsuit or drysuit. We found significant associations between the rate of change in Tc and our measured and calculated anthropometric values for each suit type in the whole group and in women, with the exception of BF% in the group wearing drysuits and BMI in female drysuit divers. These data suggest that, despite no extensive direct or indirect exposure to water while wearing a drysuit, anthropometrics also remain an important consideration when deciding how much thermal protection to wear underneath the drysuit. This stated, sex-specific associations within a suit type revealed significant associations between all anthropometric measures and calculations and the rate of change in Tc only in females in either suit type. Conversely, only BM was associated with the rate of change in Tc in males wearing drysuits. These sex-specific differences in associations with anthropometrics likely explain why we did not find any significant differences between males and females with respect to decreases in Tc despite significant differences in anthropometrics mentioned above.

Interestingly, a previous study by our group in mostly male SCUBA divers wearing thinner wetsuits while diving in warmer water (17–19 °C) revealed significant anthropometric associations with the rate of change in temperature normalized to bottom time rather than total dive time as reported here (Bradbury et al. 2024). The reasons for the discrepancy between the two studies are currently unknown but are likely multifactorial, including water temperature, dive times, and the thickness of the wetsuits used in that study. Taken together, our results suggest that BM was the only anthropometric characteristic that was significantly associated with the rate of change in Tc during the dive, except in males wearing wetsuits. Accordingly, anthropometrics should be considered an important determinant for the choice to wear a wetsuit or drysuit when SCUBA diving in ~ 10 °C water.

### Limitations

The telemetric pills used for this study do not continuously record temperature, so there was no continuous measurement of temperature change between groups. Thus, the rate of change in Tc at various points throughout the dive may have differed between groups and been undetected.

With respect to our anthropometric associations, we used a calculated BF% rather than a direct measure of BF%. Direct measures may have resulted in either significant or stronger associations with changes in Tc. Similarly, our significant sex-specific associations within each suit type had R^2^ values ranging from 0.36 to 0.60 meaning that something else explains 64 to 40% of the variability in the rate of change in Tc observed in our divers. Other factors that could contribute to the rate of change in Tc seen in the divers include, but are not limited to, differences in heat production or heat conservation mechanisms we were not able to measure in this study including the contribution of non-shivering thermogenesis or differences in the cutaneous vasoconstrictor response to the cold. In the group of females, it is possible that variability in menstrual cycle phase or sex hormone concentrations may have contributed to differences in the rate of change in Tc/min in the groups of females. However, none of these variables were measured or tracked in the current study; therefore, future investigations should consider including these measurements, when feasible, into their study designs.

The drysuit divers in the present study have years of experience and dive more frequently than those wearing wetsuits in the same waters in which the study was conducted. Therefore, they are exposed to cold water conditions more frequently than beginners. Thus, participants wearing drysuits may have a greater degree of cold acclimation than those wearing wetsuits. Their experience level could have altered thermoregulation, as more experienced divers, in theory, undergo less energy expenditure during their dives than novices. In addition, older individuals have compromised thermoregulation compared to younger divers, which could have driven temperature decreases in drysuit wearers (Kenney and Munce 2003). However, the mean age of drysuit divers was relatively low (35.8 ± 11.9, Table 3), and the age-related reductions in thermogenesis and cold-induced vasoconstriction have been primarily reported in the age range of 50–70 years (Kenney and Munce 2003). Taken together, the ages of our drysuit participants were not likely a significant driver in thermoregulation in the present study, as they did not have a substantial reduction in temperature (Table 3). More experienced divers would be expected to waste less air on buoyancy control. However, instructors and divemasters often carry additional weight and other peripherals for emergencies and training, which would require additional air to establish neutral buoyancy initially. This stated, there was no significant difference between the two suit groups for air consumption, suggesting that all groups consumed approximately the same amount of gas per minute.

It is possible that the estimated minute ventilation calculated in the present study did not accurately reflect relative effort from participants, as it was not standardized for anthropometrics. In addition, air consumption could not be separated between that which is used for breathing compared with what is used for buoyancy control or air sharing. Nevertheless, within a suit type, we did not observe differences in air consumption between males and females; any potential variability was not likely influenced by differences in effort (Table 1). In addition, there is some degree of inaccuracy when utilizing mechanical pressure gauges (SPGs) to determine pre-dive vs. post-dive tank pressure. These gauges are read manually through an analog gauge and require the researcher to round to the nearest whole number as they display pressure. Variability in tank pressure measurements could have subsequently impacted the divers’ ventilation (Table 1).

### Practical considerations

From a practical perspective, the US Navy has determined that a 1 °C reduction in core temperature is the acceptable limit (Beatty and Berghage 1972; Doubt 1996). Thus, a decrease in Tc of 0.02 °C per minute of dive time would result in a reduction of 1 °C with a 50-min dive, with greater decreases in temperature leading to shorter dives by necessity to keep Tc within the 1 °C US Navy exposure limits.

Data from our study would suggest that most of our subjects wearing drysuits with their preferred insulation kept their temperature in the thermoneutral range (− 0.02 and + 0.02 °C per minute of dive time) to avoid reaching US Navy exposure limits. Nevertheless, the associations between the rate of change in Tc and BM were the strongest in the drysuit data set. In females wearing a drysuit, all but one woman with a body mass less than or equal to 70 kg experienced a decrease in body temperature. In males wearing a drysuit, all but one man with a BM greater than or equal to 80 kg kept their Tc from decreasing during the dive. These potential cutoffs for males and females may be helpful for those divers determining whether to use additional insulation when diving in drysuits in ~ 10 °C water.

Similarly, in those wearing wetsuits with 14 mm in the core and 7 mm in the extremities, only those females with a BM less than or equal to 70 kg and males with a BM equal to or less than 80 kg experienced a decrease in Tc equal to or greater than 0.02 °C per minute of dive time. As mentioned above, these rates of change would likely equal or exceed the US Navy’s exposure limits for a 50-min dive. As above, these potential cutoffs for males and females may be helpful for those divers determining whether to use additional layers of neoprene insulation when diving in wetsuits in ~ 10 °C water and/or requiring a drysuit to maintain core temperature within exposure limits.

Of note, these guidelines should not be considered hard cut-off values, and additional confirmatory studies would be helpful to contribute to this area of research.

## Summary and conclusion

The primary findings of this study were threefold. First, we found that regardless of sex, those wearing a wetsuit had a greater decrease in Tc per minute compared to those wearing drysuits. Second, there were no sex differences in the rate of change of Tc nor Ts between males and females, regardless of suit type. Third, we found that the rate of change in Tc within a suit type while diving in ~ 10 °C water was associated with anthropometric characteristics (i.e., BSA, BM, BSA/BM ratio), whether wearing a wetsuit or a drysuit. Conversely, calculated BF% was only significantly associated with the rate of change in Tc in those wearing a wetsuit. When sex-specific anthropometric associations were examined within a suit type, we found that significant associations with the rate of change in Tc remained in females wearing either a drysuit or a wetsuit, except with BMI in females wearing a drysuit, but only BM was associated with the rate of change in Tc in males wearing a drysuit. From these data, we conclude that within each suit type, more favorable anthropometrics (i.e., lower BSA/BM ratios, and greater BMI, BM, and BF% (in wetsuit only)) were associated with smaller changes or even increases in Tc while diving. That said, the females were the driving force behind the strengths of these associations. Future studies should use temperature sensing measures for Tsk and Tc that record continuously to determine instantaneous changes in Tsk and Tc between various suit types and degrees of thermal protection from differences in wetsuit thicknesses and various forms of undergarments under multiple water temperatures.

## Data Availability

The datasets generated during and/or analysed during the current study are available from the corresponding author on reasonable request.
